# Gratitude Moderates the Mediating Effect of Deliberate Rumination on the Relationship Between Intrusive Rumination and Post-traumatic Growth

**DOI:** 10.3389/fpsyg.2019.02665

**Published:** 2019-12-03

**Authors:** Eunseung Kim, Sungman Bae

**Affiliations:** ^1^Department of Psychology, Dankook University, Cheonan, South Korea; ^2^Department of Psychology and Psychotherapy, College of Health Sciences, Dankook University, Cheonan, South Korea

**Keywords:** post-traumatic growth, intrusive rumination, deliberate rumination, gratitude, trauma

## Abstract

**Purpose:**

This study examines the moderating effect of gratitude on the mediating effect of deliberate rumination on the relationship between intrusive rumination and post-traumatic growth (PTG).

**Methods:**

We used self-report questionnaires to collect data from 450 18–68-year-old participants (M_age_ = 39.73, SD = 13.73) residing in major cities and regions across South Korea. Data that were collected from 411 participants were subjected to analysis. Version 25 of SPSS (Statistical Package for Social Science) and PROCESS macro were used to analyze mediation, moderation, and moderated mediation effects.

**Results:**

Deliberate rumination mediated the relationship between intrusive rumination and PTG. Gratitude moderated the effect of deliberate rumination on PTG. Finally, gratitude moderated the mediating effect of deliberate rumination on the relationship between intrusive rumination and PTG.

**Conclusion:**

Deliberate rumination and gratitude facilitate PTG for traumatized adults. In particular, gratitude reinforces the effect of deliberate rumination on PTG.

## Introduction

Nationwide surveys indicate that 71.9% of Korean (Seo et al., 2012) adults and 89% of American adults (Kilpatrick et al., 2013) experience at least one traumatic experience during their lifetime. Traumatic events can cause serious psychological problems (Christopher, 2004), and approximately 10–15% of those who experience trauma are diagnosed with post-traumatic stress disorder (Green and Lindy, 1994).

While past studies have focused on clinical symptoms (e.g., reexperiencing symptoms, hyperarousal) that result from the experience of a traumatic event, recent research has begun to focus on positive changes and growth that occur after a traumatic experience (Joseph et al., 1993; Nerken, 1993; Tedeschi and Calhoun, 1995). Indeed, researchers have found that PTG can occur after exposure to various types of traumatic events such as bereavement (Taku et al., 2008), cancer (Cormio et al., 2017), physical violence (Kleim and Ehlers, 2009), and traffic accidents (Nishi et al., 2010).

Post-traumatic growth refers to the positive changes and growth that occur as a result of experiencing trauma (Tedeschi and Calhoun, 2004). PTG can lead to an enhanced sense of personal strength, changes in one’s perspective toward life, and improved interpersonal relationships. Some individuals experience positive changes and growth after trauma, although others experience intrusive symptoms such as nightmares, hypo/hyperarousal, depression, and negative changes in cognition and mood. Persistent depression is linked to worse outcomes such as maladaptive coping and maladjustment (Ghio et al., 2015).

Why do some people experience PTG? According to previous studies, factors related to PTG can be classified into three categories: the characteristics of the event, personal characteristics, and cognitive processing of the traumatic experience (Calhoun et al., 2000; Linley and Joseph, 2004; Tedeschi and Calhoun, 2004; Kim and Lee, 2016). In particular, many researchers have focused on the ways in which individuals cognitively process a traumatic event (e.g., intrusive and deliberate rumination).

Rumination plays a key role in the process of PTG and is divided into two categories: intrusive and deliberate rumination (Calhoun and Tedeschi, 2006). Intrusive rumination is a process by which one automatically reexperiences images, emotions, and thoughts that are related to an event. On the other hand, deliberate rumination refers to an intentional thought process through which one attempts to understand the cause and meaning of an incident. After a traumatic event, affected individuals may experience intrusive rumination and extreme emotional distress, but they may also simultaneously attempt to engage in deliberate rumination to alleviate psychological distress (Wu et al., 2015; Zhou and Wu, 2015; Zhang et al., 2018).

Deliberate rumination can facilitate the expansion of existing schemas and help one better understand a traumatic experience (Triplett et al., 2012). An individual’s beliefs and values may be changed and enriched through the process of deliberate rumination, and this in turn may promote PTG. PTG can lead to various positive changes such as an increased appreciation of life and self-understanding as well as changes in life priorities (Calhoun et al., 2010).

Tedeschi and Calhoun (1996, 2004) noted that the process of PTG occurs across several stages. Individuals possess a schema that corresponds to a cognitive framework of the self, others, and the world. After traumatic events, affected individuals experience distortions of their belief systems, and intrusive rumination is activated in response to extreme psychological stress (Zhou and Wu, 2015; Zhang et al., 2018). However, intrusive thoughts may activate deliberate rumination, which may help individuals better understand their traumatic experience and rebuild a new schema (Triplett et al., 2012; Tsai et al., 2016; Gwak and Park, 2018).

Deliberate rumination is an attempt to accommodate trauma into one’s cognitive schema. It is an active cognitive process that involves reconstructing an existing schema to promote PTG. The effects of deliberate rumination on PTG have been demonstrated in the literature (Tedeschi and Calhoun, 2004, Calhoun and Tedeschi, 2006), and higher levels of deliberate rumination have been found to be associated with greater PTG (Linley and Joseph, 2004; Taku et al., 2008; Triplett et al., 2012; Zhou and Wu, 2015; Cárdenas et al., 2019). In particular, Triplett et al. (2012) found that deliberate rumination mediates the relationship between intrusive rumination and PTG.

Gratitude has been identified as a factor that promotes PTG. Gratitude refers to an attitude of thankfulness and joy for the benefits and blessings that one has received from other people and nature (Emmons and Shelton, 2002). Adler and Fagley (2005) noted that gratitude involves “noticing and acknowledging its value and meaning of something—an event, behavior, object—and feeling a positive emotional connection to it.” Gratitude includes cognitive, emotional, and behavioral factors (McCullough et al., 2001; Watkins et al., 2003; Adler and Fagley, 2005) and is an attribution-dependent characteristic (Weiner, 1985) that allows one to perceive the benefits that have been gained from others and various life experiences (Emmons, 2007). Gratitude consists of an appreciation of others and various aspects of daily life and the ability to recall positive past experiences (Watkins et al., 2003).

A recent meta-analytic study found that gratitude is a significant predictor of PTG (Jang and Kim, 2017). Recent studies have also shown that gratitude moderates the effect of post-traumatic stress on PTG (Vieselmeyer et al., 2017; Leppma et al., 2018). Highly grateful individuals tend to be more likely to appreciate everyday events (McCullough et al., 2002), which in turn can facilitate adaptive coping (Fredrickson, 2004). Such individuals experience gratitude more frequently in daily life and across a wider array of circumstances compared to those with lower levels of gratitude. Further, experiences of gratitude in the midst of trauma may be significant because gratitude not only inspires and transforms individuals, it also offers meaning in life by helping people interpret their life as a gift. Gratefulness may also have long-term survival benefits by making people more open-minded and flexible, ultimately allowing them to better perceive and take advantage of opportunities, which in turn can facilitate adaptive coping (Johnson and Fredrickson, 2005). Past studies have also shown that gratitude promotes deliberate rumination, which promotes PTG (Wood et al., 2010; Zhou and Wu, 2015; Kim and Lee, 2016).

In particular, Zhou and Wu (2015) longitudinally examined the relationship between gratitude, deliberate rumination, and PTG among people who had experienced an earthquake. Specifically, the participants were assessed three and a half years (T1), four and a half years (T2), and five and a half years (T3) after the earthquake. The results showed that gratitude predicted PTG from T2 and T3. This result indicates that gratitude is a stable predictor of PTG. Additionally, gratitude at T1 predicted PTG at T3 through deliberate rumination at T2.

**FIGURE 1:**
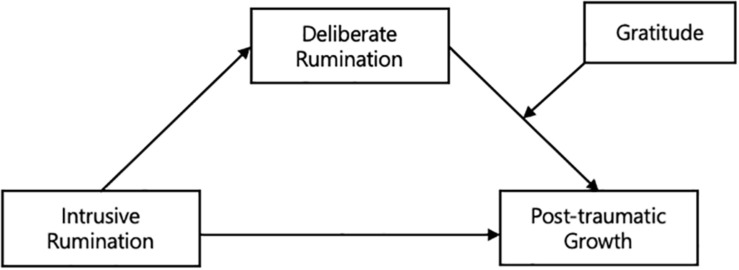
Hypothetical Model.

In summary, the mediating effect of deliberate rumination on the relationship between intrusive rumination and PTG has been established in the literature. In the present study, gratitude was expected to moderate the relationship between deliberate rumination and PTG. Accordingly, five research hypotheses were formulated (Figure 1).

Hypothesis 1: Intrusive rumination will be negatively related to PTG.

Hypotheses 2: Deliberate rumination will mediate the relationship between intrusive rumination and PTG.

Hypothesis 3: Gratitude will be positively related to PTG.

Hypothesis 4: Gratitude will moderate the relationship between deliberate rumination and PTG.

Hypothesis 5: The mediating effect of deliberate rumination on the relationship between intrusive rumination and PTG will be moderated by gratitude.

## Materials and Methods

### Participants and Procedures

Participants in this study were recruited from community centers in major cities and regions across South Korea. Trained investigators (e.g., psychology graduate students) provided participants with a detailed introduction to the study. All participants provided written informed consent in accordance with the Declaration of Helsinki and signed a consent form before completing the questionnaires. After the participants completed the questionnaires, they were compensated. This study was approved by the Institutional Review Board of Dankook University.

This study was conducted among 450 adults with an age range of 19–68 years; the mean age of participants was 39.73 years (SD = 13.73). The sample consisted of 245 women (59.6%) and 166 men (40.4%). The age distribution of the participants was as follows: 123 (29.9%), 98 (23.8%), 83 (20.2%), 69 (16.8%), and 38 (9.2%) participants were in their 20s, 30s, 40s, 50s, and 60s, respectively. Further, 155 (37.7%), 105 (25.3%), 56 (10.6%), 41 (10.0%), and 53 (12.9%) participants were college, graduate school, university, professional college, and high school graduates, respectively; 0.5% had no educational experience. Additionally, 230 (56%) and 163 (36.7%) participants were married and unmarried, respectively (Table 1). Finally, using Allen’s (2005) categorization of trauma into three types – interpersonal, impersonal, and interpersonal related trauma – 143 (34.8%) participants of this study experienced interpersonal trauma inflicted by human perpetrators (e.g., physical or sexual abuse), 146 (35.5%) experienced impersonal trauma caused by human or natural origins (e.g., accidents or natural disasters), and 122 (29.7%) experienced interpersonal related trauma (e.g., death of a partner, severe illness).

**TABLE 1:** Demographic Information.

***N* = 411**			
		**Frequency**	**percent**
Sex	Male	166	40.4%
	Female	245	59.6%
Age	20–29	123	29.9%
	30–39	98	23.8%
	40–49	83	20.2%
	50–59	69	16.8%
	60–69	38	9.2%
Education	No experience	2	0.5%
	Primary School and Middle school	53	12.9%
	Community college graduate	41	10.0%
	College undergraduate	56	13.6%
	College graduation	155	37.7%
	Graduate grad school	105	25.3%
Marriage	Single	163	39.7%
	Marriage	230	56.0%
	Divorced	14	3.4%
	Bereavement	4	1.0%
Income Status	High	5	1.2%
	Middle-high	83	20.2%
	Middle	210	51.1%
	Middle-low	94	22.9%
	Low	19	4.6%
Religious	No religion	159	38.7%
	Catholic	26	6.3%
	Protestant	197	47.9%
	Buddhism	28	6.8%
	Won Buddhism	1	0.2%
			

### Measures

#### Trauma Experience Questionnaire

In the present study, the Trauma Experience Questionnaire, which was developed by Song et al. (2009) and revised by Shin and Chung (2012), was used to obtain information about traumatic events participants had experienced (e.g., type, duration, and severity of the traumatic event). The questionnaire contained seven items. Participants were asked to disclose the most painful traumatic event that they had experienced and to categorize the type of the event. They were then instructed to respond to the items of the assessment based on the aforementioned event. They reported whether they experienced psychological pain and scored the severity of the subjective pain that they had experienced at the time of the event and more recently on a scale of 1–7 (1 = no pain; 7 = very painful).

#### Korean Version of the Event-Related Rumination Inventory

The Korean version of the Event-Related Rumination Inventory was developed by Cann et al. (2011) and revised by Ahn et al. (2013). The scale consists of 20 items that assess intrusive and deliberate rumination using a 4-point Likert scale (0 = not at all; 3 = always). A few examples of items that assess intrusive rumination are as follows: “Thoughts, memories, or images of the event came to mind even when I did not want them” and “I could not keep images or thoughts about the event from entering my mind.” A few examples of items that assess deliberate rumination are as follows: “I thought about whether I could find meaning from my experience,” “I thought about the event and tried to understand what happened,” and “I deliberately thought about how the event had affected me.” In the present study, the Cronbach’s alphas of the intrusive and deliberate rumination subscales were 0.93 and 0.91, respectively.

#### Korean Version of the Post-traumatic Growth Inventory

The Korean version of the Post-Traumatic Growth Inventory that was developed by Tedeschi and Calhoun (1996) and validated by Song et al. (2009) was used to measure individual perceptions of positive changes after a traumatic experience. The scale consists of 21 items with four subscales: relating to others, new possibilities, personal strength, and spiritual change. For each of the subscales, responses are recorded on a 6-point Likert scale (0 = no change; 5 = very high degree of change). Higher total scores are indicative of greater PTG. The following are representative scale items: “I changed my priorities about what is important in my life,” “I established a new path for my life,” and “I am more likely to try to change things that need changing.” The Cronbach’s alpha of this scale was 0.94 in the present study.

#### Korean Version of the Gratitude Questionnaire

We used the Korean version of the Gratitude Questionnaire that was developed by McCullough et al. (2002) and validated by Kwon et al. (2006). It measures the intensity, frequency, extent, and density of gratitude, consisting of 6 items that require responses to be recorded on a 7-point Likert scale (0 = strongly disagree; 6 = strongly agree). The following are representative scale items: “I have so much in life to be thankful for” and “As I get older, I find myself better able to appreciate the people, events, and situations that have been part of my life.” Two items are reverse-scored, and higher total scores are indicative of greater gratitude. The Cronbach’s alpha of this scale was 0.89 in the present study.

### Data Analysis

Version 25.0 of SPSS and PROCESS macro 2.16 for SPSS (Hayes, 2013) were used to test the research hypotheses. First, frequencies and descriptive statistics were computed to examine the demographic characteristics of the sample, and Pearson’s correlation analysis was conducted to examine the relationships between the study variables. Second, model 4 of PROCESS macro for SPSS was used to examine the mediating effect of deliberate rumination on the relationship between intrusive rumination and PTG (Zhao et al., 2010; Rucker et al., 2011). Next, a bootstrap test was conducted and the resultant 95% confidence intervals were inspected to examine the significance of the indirect effects that resulted from mediation analysis (Preacher et al., 2007).

Model 1 of PROCESS macro for SPSS was used to examine the moderating effect of gratitude on the relationship between deliberate rumination and PTG. Additionally, simple regression was conducted to examine the effect of deliberate rumination on PTG as a function of the level of gratitude (i.e., high vs. low). Finally, model 14 of PROCESS macro for SPSS was used to examine whether gratitude moderates the mediating effect of deliberate rumination on the relationship between intrusive rumination and PTG.

## Results

### Correlation Analysis

Emergent correlations between the study variables are shown in Table 2. Intrusive rumination correlated positively with deliberate rumination (*r* = 0.36, *p* < 0.01) and negatively with gratitude (*r* = −0.29, *p* < 0.01). On the other hand, deliberate rumination was unrelated to PTG. Deliberate rumination correlated positively with PTG (*r* = 0.36, *p* < 0.01) but was unrelated to gratitude. Finally, there was a positive correlation (*r* = 0.45, *p* < 0.01) between gratitude and PTG.

**TABLE 2:** Correlations, mean, and standard deviation among variables (*N* = 411).

	**Intrusive rumination**	**Deliberate rumination**	**Gratitude**	**Post-traumatic growth**
Intrusive rumination				
Deliberate rumination	0.36^∗∗^			
Gratitude	–0.29^∗∗^	0.02		
Post-traumatic growth	–0.03	0.36^∗∗^	0.45^∗∗^	
Mean	17.94	22.98	34.95	79.47
SD	6.97	7.01	5.96	22.60

*^∗∗^*P* < 0.01.*

### The Mediating Effect of Deliberate Rumination on the Relationship Between Intrusive Rumination and PTG

The mediating effect of deliberate rumination on the relationship between intrusive rumination and PTG was examined (Table 3). Intrusive rumination had a negative effect on PTG (*B* = 0.60, *t* = −3.80, *p* < 0.001) and a positive effect on deliberate rumination (*B* = 0.37, *t* = 7.91, *p* < 0.001). Deliberate rumination had a positive effect on PTG (*B* = 1.36, *t* = 8.77, *p* < 0.001). In addition, the mediating effect of deliberate rumination on the relationship between intrusive rumination and PTG was statistically significant (95% confidence interval = 0.36, 0.67) (Table 4).

**TABLE 3:** Mediating effects of deliberate rumination in relationship between intrusive rumination and post-traumatic growth).

**DV**	**IV**	**B**	**S.E**	***t***	***p***	**95% CI**		***F***	***R* ^2^**
						**Lower**	**Upper**		
Deliberate rumination	(constant)	16.33	0.90	18.10	0.00	14.56	18.10	62.51^∗∗∗^	0.13
	Intrusive rumination	0.37	0.05	7.91	0.00	0.28	0.46		
Post-traumatic growth	Intrusive rumination	0.60	0.16	–3.80	0.00	–0.91	–0.29	38.63^∗∗∗^	0.16
	Deliberate rumination	1.36	0.16	8.77	0.00	1.06	1.67		

*^∗∗∗^*P* < 0.001.*

**TABLE 4:** Indirect effects on post-traumatic growth (Bootstrapping).

**Pathway**	**Coefficient**	**SE**	**95% CI**	
			**Lower**	**Upper**
Intrusive rumination → Deliberate rumination → Post-traumatic growth	0.50	0.08	0.36	0.67

### The Moderating Effect of Gratitude on the Relationship Between Deliberate Rumination and PTG

The moderating effect of gratitude was examined by entering deliberate rumination, gratitude, and their interaction terms (i.e., deliberate rumination × gratitude) into the model. Table 5 shows the results of the analysis that was conducted to examine the moderating effect of gratitude on the relationship between deliberate rumination and PTG.

**TABLE 5:** The moderating effects of gratitude on the relationship between intentional rumination and post-traumatic growth).

**DV**	**IV**	**B**	**S.E**	**t**	**p**	**95% CI**		***F***	***R* ^2^**
						**Lower**	**Upper**		
Post-traumatic growth	(constant)	79.50	0.92	86.79	0.00	77.20	81.30	66.79^∗∗∗^	0.33
	Deliberate rumination	1.12	0.13	8.55	0.00	0.85	1.36		
	Gratitude	1.61	0.16	10.27	0.00	1.30	1.91		
	Deliberate rumination × Gratitude	–0.04	0.02	–2.10	0.03	–0.08	–0.01		

*^∗∗∗^*p* < 0.001.*

**FIGURE 2:**
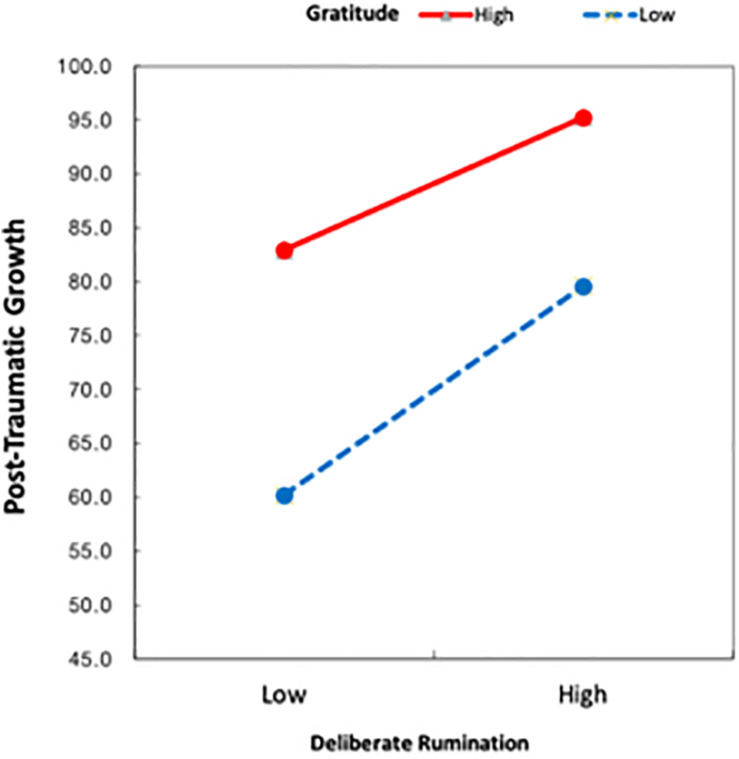
Gratitude moderated the relationship between deliberated rumination and post-traumatic growth.

The main effects of deliberate rumination (*B* = 1.12, *t* = 8.55, *p* < 0.001) and gratitude (*B* = 1.61, *t* = 10.27, *p* < 0.001) as well as their interaction effect (i.e., deliberate rumination × gratitude) (*B* = −0.04, *t* = −2.10, *p* < 0.05) were statistically significant. Simple regression analysis was conducted to examine the effect of deliberate rumination on PTG as a function of the level of gratitude (Table 6). The results revealed that the 95% confidence intervals did not include a 0. In other words, the effect of deliberate rumination on PTG differed between individuals who reported low (i.e., M − 1 SD) and high (i.e., M + 1 SD) levels of gratitude (Figure 2). Gratitude reinforced the effect of deliberate rumination on PTG.

**TABLE 6:** Conditional indirect effect at specific levels of the gratitude when deliberate rumination as a mediator.

**Conditional effect of gratitude**	**Estimate**	**S.E**	**t**	**p**	**95% CI**	
					**Lower**	**Upper**
−1SD	–5.95	0.17	7.99	0.00	1.02	1.68
M	1.05	0.13	8.06	0.00	0.80	1.32
+1SD	0.86	0.18	4.83	0.00	0.51	0.1.21

### The Moderating Effect of Gratitude on the Mediating Effect of Deliberate Rumination on the Relationship Between Intrusive Rumination and PTG

**FIGURE 3:**
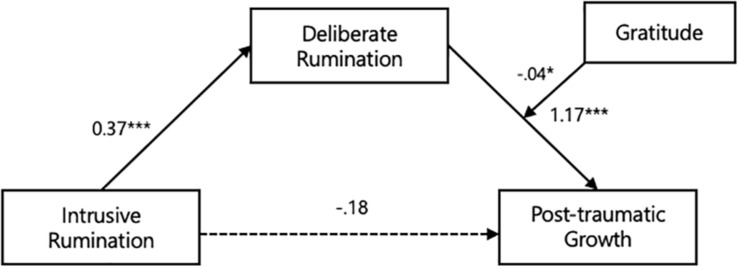
Gratitude moderated the mediating effect of deliberate rumination between intrusive rumination and post-traumatic growth. ^∗^*p* < 0.05, ^∗∗^*p* < 0.01, ^∗∗∗^*p* < 0.001.

Table 7 shows the results of the analysis that was conducted to examine the moderating effect of gratitude on the mediating effect of deliberate rumination on the relationship between intrusive rumination and PTG. The direct effect of intrusive rumination on PTG was not significant (*B* = −0.18, *t* = −1.19, *p* = 0.23). However, deliberate rumination had a positive effect on PTG (*B* = 1.17, *t* = 3.88, *p* < 0.001), the interaction term (deliberate rumination × gratitude) (*B* = −0.04, *t* = −2.25, *p* < 0.05) was significant, and the index of the moderated mediation effect was significant (95% confidence interval = −0.033, −0.002) (Figure 3). Further, Table 8 shows the results of the bootstrap test, which revealed the conditional indirect effect of gratitude. The 95% confidence intervals did not include a 0. In other words, the moderating effect of gratitude through deliberate rumination on PTG differed for individuals who reported low (i.e., M − 1 SD), average (i.e., mean), and high (i.e., M + 1 SD) levels of gratitude.

**TABLE 7:** Moderated mediation effects of gratitude on the relationship between intrusive rumination, deliberate rumination, and post-traumatic growth.

	**Mediator variables models (DV: Deliberate rumination)**								
	***B***	**S.E**	***t***	*p*	**Lower**	**Upper**
(constant)	–0.0005	0.33	–0.0017	0.9987	–0.6421	0.6410
Intrusive rumination	0.37	0.05	7.91	0.00	0.28	0.46

	**Moderator variable models (DV: Post-traumatic growth)**								
	***B***	**S. E**	***t***	***p***	**Lower**	**Upper**

(constant)	0.03	0.92	0.04	0.97	–1.76	1.84
Intrusive rumination	–0.18	0.15	–1.19	0.23	–0.47	0.12
Deliberate rumination	1.17	0.14	8.36	0.00	0.90	1.45
Gratitude	1.54	0.16	9.29	0.00	1.21	1.87
Deliberate rumination × Gratitude	–0.04	0.02	–2.25	0.03	–0.08	–0.01
Moderate mediation index	S.E		Lower		Upper	
−0.016	0.008		−0.033		−0.002	

**TABLE 8:** Conditional indirect effect of gratitude when deliberate rumination mediated between intrusive rumination and post-traumatic growth.

**Mediator**	**Gratitude**	**Estimates**	**S.E**	**95% CI**	
				**Lower**	**Upper**
Deliberate rumination	−1SD	0.53	0.09	0.36	0.73
	M	0.42	0.07	0.29	0.56
	+1SD	0.34	0.07	0.20	0.49

## Discussion

The major findings of the present study are discussed in this section. The previously observed mediating effect of deliberate rumination on the relationship between intrusive rumination and PTG was verified. Intrusive rumination facilitated PTG by promoting deliberate rumination (Taku et al., 2008; Triplett et al., 2012; Wu et al., 2015). These results are consistent with past findings that post-traumatic intrusive rumination stimulates deliberate rumination, which in turn promotes PTG (Triplett et al., 2012; Tsai et al., 2016; Zhou and Wu, 2016; Zhang et al., 2018).

The results of this study support the PTG model that was proposed by Tedeschi and Calhoun (2004). According to this model, deliberate rumination plays a key role in the PTG process. Many individuals experience intrusive rumination after traumatic events (Scarpa et al., 2009), and intrusive rumination can activate deliberate rumination. Persistent intrusive rumination can adversely impact PTG and is likely to cause post-traumatic stress disorder (Triplett et al., 2012). In particular, deliberate rumination can help one reconstruct an incident and correct dysfunctional beliefs about a traumatic event (Seo and Chae, 2006).

Second, in the present study, gratitude had a positive impact on PTG, and deliberate rumination and gratitude had an interaction effect on PTG. These results concur with past findings that the influence of deliberate rumination on PTG is strengthened by high levels of gratitude (Zhou and Wu, 2015; Kim and Lee, 2016). This suggests that the effect of deliberate rumination on PTG is reinforced by gratitude.

Past studies have only examined the direct relationship between gratitude and PTG. In this regard, the present findings further our understanding of the specific role that gratitude plays in PTG by delineating the moderating effect of gratitude on the relationship between deliberate rumination and PTG. Gratitude can alter the perspectives from which traumatic experiences are interpreted (Watkins, 2014). Highly grateful individuals tend to find positive resources in their lives after a traumatic event and perceive themselves and their environments positively (Fredrickson, 2004). In other words, gratitude can help individuals find new meaning and value after a traumatic experience and accept painful experiences as a part of their lives.

One of the key findings of this study pertains to the moderating effect of gratitude on the mediating effect of deliberate rumination on the relationship between intrusive rumination and PTG. This result is consistent with past findings that highly grateful individuals experience greater PTG as a result of their cognitive efforts to understand a traumatic event (McCullough et al., 2006; Chun and Lee, 2013; Watkins, 2014). This suggests that the impact of deliberate rumination on PTG varies as a function of the level of gratitude. In this regard, gratitude can activate deliberate rumination and act as a buffer against the psychological distress that is caused by intrusive thoughts (Tsai et al., 2016; Leppma et al., 2018).

This study makes several contributions to the literature. First, past studies that have examined the impact of gratitude on PTG have been conducted using developmentally homogeneous samples such as adolescents and middle-aged adults. However, in this study, we used a heterogeneous sample of participants who represented all the developmental stages ranging from youth to older adulthood. This enhances the generalizability of the observed effect of gratitude on PTG to people of all ages. Second, the association between the gratitude and PTG has been observed in previous studies, but the moderating effect of gratitude on PTG has not been clearly delineated. In addition, there has been a lack of understanding about how deliberate rumination contributes to PTG. In this study, we have delineated the moderating effect that gratitude has on the mediating effect of deliberate rumination on the relationship between intrusive rumination and PTG.

The present findings have clinical implications. Specifically, therapists must be informed that repetitive and intrusive thoughts are natural reactions to a traumatic event and that traumatized individuals do not need to excessively suppress or avoid thoughts about their traumatic experience. In addition, it is necessary to implement training and intervention programs that can help individuals engage in deliberate rather than intrusive rumination. In particular, the present finding that gratitude enhances the effect of deliberate rumination on PTG suggests that psychological interventions should aim to promote gratitude among traumatized individuals (e.g., gratitude writing).

This study has several limitations. First, self-report measures were used to collect data in the present study; thus, future studies should use behavioral observations and the reports of family members and acquaintances to measure the study variables. Second, as this study used a cross-sectional research design, inferences cannot be drawn about the causality of emergent relationships. Future studies should use a longitudinal research design to test the validity of the present findings. Third, we did not distinguish between different types of trauma and the period after trauma occurrence; we weighed all traumatic experiences equally, although research suggests that different types of trauma and the period after occurrence might differently influence PTG.

Despite its limitations, this is the first study to assess the moderating effect of gratitude on the mediating effect of deliberate rumination on the relationship between intrusive rumination and PTG. These findings shed light on how and when gratitude is associated with PTG via deliberate rumination. Further, this study provides grounds for gratitude interventions for adults who have experienced trauma to facilitate growth. It also implies that in the midst of distress, those who have experienced trauma may be able to consider their life more meaningful through gratitude.

## Data Availability Statement

The datasets generated for this study are available on request to the corresponding author.

## Ethics Statement

All subjects gave written informed consent in accordance with the Declaration of Helsinki. This study was approved by the Institutional Review Board of Dankook University.

## Author Contributions

EK collected and analyzed the data and wrote the first draft of the manuscript. SB led manuscript writing and revised it critically for important content. Both authors participated in the final approval of the version to be published and agreed to be accountable for all aspects of the work.

## Conflict of Interest

The authors declare that the research was conducted in the absence of any commercial or financial relationships that could be construed as a potential conflict of interest.

## Abbreviations

PTG: post-traumatic growth
SPSS: Statistical Package for Social Science.
